# Upper-body morbidity following breast cancer treatment is common, may persist longer-term and adversely influences quality of life

**DOI:** 10.1186/1477-7525-8-92

**Published:** 2010-08-31

**Authors:** Sandra C Hayes, Sheree Rye, Diana Battistutta, Tracey DiSipio, Beth Newman

**Affiliations:** 1 School of Public Health, Queensland University of Technology, Victoria Park Road, Kelvin Grove, Queensland, 4059, Australia; 2 Institute of Health and Biomedical Innovation, Queensland University of Technology, Victoria Park Road, Kelvin Grove, Queensland, 4059, Australia

## Abstract

**Background:**

Impairments in upper-body function (UBF) are common following breast cancer. However, the relationship between arm morbidity and quality of life (QoL) remains unclear. This investigation uses longitudinal data to describe UBF in a population-based sample of women with breast cancer and examines its relationship with QoL.

**Methods:**

Australian women (n = 287) with unilateral breast cancer were assessed at three-monthly intervals, from six- to 18-months post-surgery (PS). Strength, endurance and flexibility were used to assess objective UBF, while the Disability of the Arm, Shoulder and Hand questionnaire and the Functional Assessment of Cancer Therapy-Breast questionnaire were used to assess self-reported UBF and QoL, respectively.

**Results:**

Although mean UBF improved over time, up to 41% of women revealed declines in UBF between six- and 18-months PS. Older age, lower socioeconomic position, treatment on the dominant side, mastectomy, more extensive lymph node removal and having lymphoedema each increased odds of declines in UBF by at least two-fold (p < 0.05). Lower baseline and declines in perceived UBF between six- and 18-months PS were each associated with poorer QoL at 18-months PS (p < 0.05).

**Conclusions:**

Significant upper-body morbidity is experienced by many following breast cancer treatment, persisting longer term, and adversely influencing the QoL of breast cancer survivors.

## Background

Whether as a consequence of earlier detection methods and/or advances in treatment, survival following breast cancer is among the best of any cancer [1]. Good survival prospects contribute to the significant and growing number of breast cancer survivors, making quality of survival an important personal and public health issue warranting research attention.

Work published more than a decade ago highlighted that between 15-45% of breast cancer survivors reported impairments in upper-body function (UBF) up to one year post-surgery, depending on timing of assessment and the particular impairment [2-4]. While impaired arm function was short-lived for some, a subgroup of women reported persisting problems longer term [5].

Since this work, the majority of published literature describing UBF following breast cancer compares outcomes of treatment techniques (e.g., sentinel node biopsy versus axillary dissection; breast-conserving surgery versus mastectomy; radiation to the chest wall and axilla versus to the axilla only) [6-11]. The studies tend to be retrospective and are subject to the greater potential for selection bias associated with clinic- rather than population-based samples [12-14]. Further, the literature relies on self-reported measures of UBF, typically in the absence of clinical assessment. These design limitations are critical: lack of longitudinal data compromises generalisability of study results; focus on treatment outcomes has overshadowed the potential impact of other personal and/or behavioural characteristics; and previous work has demonstrated that correlations between objective and self-reported measures of UBF are only modest (r = -0.2-0.3) indicating they reflect different constructs, with both the direction and magnitude of relationships with other characteristics varying depending on how UBF is measured [15]. For example, those treated on the dominant side show better UBF when assessed clinically, but report poorer perceived function, when compared with those treated on their non-dominant side.

A systematic review, published in 2003 [16], was undertaken to describe the relationship between upper-body morbidity and quality of life (QoL). The authors concluded that while arm morbidity was associated with poorer QoL, the strength of the association was considered low and only four investigations contributed to their findings. Since the review, others have demonstrated that upper-body impairments adversely affect the ability to participate in daily activities as well as QoL [17-19], and there is evidence to suggest QoL predicts survival [20]. However, the study designs of this research have limited the evaluation of a causal pathway between UBF and QoL.

This investigation prospectively describes UBF, measured objectively and self-reported, in a population-based sample of women with breast cancer between six- and 18-months post-surgery (PS). The characteristics associated with UBF and its relationships with QoL are examined.

## Methods

### Patient group

This work forms a planned component of a broader study, The Pulling Through Study, which was designed to document the prevalence and severity of problems, including UBF, between six- and 18-months following breast cancer surgery among Australian women [21]. Women aged 74 years or younger, with a first diagnosis of invasive, unilateral breast cancer, and residing within a 100 kilometre radius of Brisbane, Queensland, were eligible for participation. Eligibility criteria minimised the impact of age-related co-morbidities on study findings, allowed for the untreated side to serve as a 'control' for certain outcomes, and enabled attendance at regular clinical assessments. Younger women (< 50 years) were over-sampled to ensure adequate numbers were available for specific age group analyses. Population-based sampling was undertaken through the Queensland Cancer Registry. It takes three to four months from the point of cancer diagnosis for patient information to arrive at the registry. Consequently, recruitment procedures commenced around four months PS. Following ethical approval (Queensland University of Technology, Ref No 2179H), doctor consent was obtained for 417 women (82% of random sample). Participant consent was obtained from 71% (n = 294); thereafter, seven women decided not to participate or could not be recontacted. Hence, 287 women participated in baseline measures; the majority participated in all components of data collection (75%), while the remainder participated on a 'questionnaire-only' basis.

### Data collection

Clinical assessment of UBF and completion of patient-administered questionnaires occurred every three months, over a 12-month period, with baseline assessment at six-months PS. Disease characteristics were collected from pathology reports at the Queensland Cancer Registry. Personal characteristics (such as side of dominance and income) were self-reported, and lymphoedema status was evaluated objectively using bioimpedance spectroscopy [21].

### Objective upper-body function

Clinical assessments of UBF were conducted for strength and endurance, hand grip strength, and flexibility, in that order. Upper-body strength and endurance (UBSE) were measured using an incremental exercise protocol, with each stage lasting one minute in duration and increments made by increasing speed of movement and weight held (0.5 kilogram increments, with the first one-minute stage commencing with no weight held). The movement combined a traditional 'upright row' and 'shoulder press', but the specific range of movement was individualised for each participant and each arm. To advance levels, the participant must have maintained correct form, range of movement and speed for the entire one-minute stage. Weight (kilograms, kg) held during the last successfully completed stage, assessed separately for each arm, was recorded. More details including comparison of this technique with assessment of strength and endurance using an isokinetic dynamometer are reported elsewhere [15]. A standard calibrated hand dynamometer (TTM Original Dynamometer 100 kg, Tokyo) was used to measure isometric hand grip strength (HGS). Three maximal contractions on each side, alternating between sides to allow for rest between each contraction, were performed and the maximum score achieved for each hand was recorded. Goniometry of flexion of the shoulder was used as a measure of shoulder flexibility. The participant was asked to hold her arm by her side, palm of hand positioned medially. The testing arm was then moved upward in the sagittal plane (in the flexion direction), as far as possible, with the palm of the hand always facing medially and elbow joint extended at 180 degrees. The test was undertaken twice for each side, alternating between sides. A third measure was taken when the first two measures differed by more than five degrees. The maximum range achieved for each arm was recorded. For all objective measures, results from the treated side are presented here.

### Subjective upper-body function

The Disability of the Arm, Shoulder and Hand (DASH) questionnaire was used to assess self-reported UBF. The DASH [22] comprises 30 items and collects information about the level of difficulty experienced when performing specific tasks, the extent to which any upper-body problem interferes with normal activities, and the severity of specific upper-body symptoms. Final scores range from 0 to 100, where 0 reflects no disability (good function) and 100 reflects extensive disability (poor function).

### Quality of life

The Functional Assessment of Cancer Therapy-Breast (FACT-B+4) questionnaire was included in the self-administered survey, to provide a measure of QoL. Final scores range from 0 to 160 whereby 0 reflects low QoL and 160 reflects high QoL. This QoL tool has been widely used and extensively described, including its validity and reliability, by others [23].

### Statistical Analysis

Distributions of UBF and QoL measures were approximately Normally distributed, hence means and standard deviations (SD) were used to summarise data at each phase. Absolute change in UBF between six- and 18-months PS was calculated and used to categorise participants as experiencing declines, no change, or improvements in UBF over time. Clinically, we argue that improvement following surgery is expected, with any decline in function over time potentially important, although we acknowledge that measurement error may account for some misclassification of decliners. In a sensitivity analysis of this strict position, we also categorised decline according to changes of more than 10% and reanalysed. Binary logistic regression was used to explore characteristics associated with declines in function, while regression models utilising a general linear modelling framework were used to investigate predictive relationships between function and QoL. Final regression models presented include adjustment for all potential confounders identified by statistical and/or clinical significance (further details regarding modelling provided in Hayes et al [21]). Differences of seven units in the FACTB+4 total score [23] and odds ratios (OR) of > 2 or < 0.5 were considered a priori as potentially clinically important, while a two-tailed p < 0.05 was considered statistically significant. Means (95% confidence interval, CI) and odds ratios (95% CI) are presented for continuous and dichotomous outcomes, respectively. Underlying assumptions for these analytical techniques, including the absence of multi-collinearity, were tested and met.

## Results

Study participants had similar demographic and clinical characteristics compared with the parent sample (n = 511), as presented elsewhere [21]. In summary, average age of participants was 54 years (SD: 10), and 74% had infiltrating ductal carcinoma, 16% had infiltrating lobular carcinoma and the balance had other or mixed histological types. The majority (74%) received less invasive surgery (complete local excision versus mastectomy), had lymph node dissection (87%) with a median of 12 (range 1-47) nodes examined and 0 (range 0-39) positive nodes. Radiation was a common adjuvant therapy, received by approximately 70% of women. Approximately 40% received chemotherapy and 60% received hormone therapy. Overall, approximately 30%, 48% and 17% of women received one, two or all of these forms of adjuvant therapy, respectively. With the exception of hormone therapy, active chemotherapy and/or radiation therapy were completed by nine months PS for 98% of the group.

### Upper-body function

Overall, UBF improved throughout the 12-month testing period, with the majority of improvement occurring between six- and 12-months PS, irrespective of whether UBF was measured objectively or via self-report (Table 1). Depending on the UBF measure, up to 42% of women experienced UBF declines between six- and 18-months PS (Table 2). These results were insensitive to classification of declining UBF as either any decline or decline > 10%; classifications were unchanged for UBSE (data not shown) and similar for DASH (28% instead of 34% were categorised as decliners).

**Table 1:** Mean upper-body function scores between six- and 18-months post-surgery*

	Months post-surgery									
	6		9		12		15		18	
	n	Mean (SD)	n	Mean (SD)	n	Mean (SD)	n	Mean (SD)	n	Mean (SD)
*Objective measures*										
UBSE (kg)	212	0.8 (0.5)	185	0.9^‡ ^(0.6)	179	0.9^‡ ^(0.6)	169	1.0^‡ ^(0.6)	186	0.9^‡ ^(0.6)
HG Strength (kg)	215	16.1 (6.7)	190	16.3 (6.6)	187	16.6 (6.9)	174	17.3^‡ ^(7.1)	187	16.9 (7.5)
Flexibility^† ^(degrees)	215	143.0 (12.5)	191	145.7^‡ ^(11.2)	189	147.5^‡ ^(10.2)	178	148.3^‡ ^(10.4)	195	150.2^‡ ^(10.5)
*Self-report measure*										
DASH score	258	14.2 (14.2)	256	13.0^‡ ^(14.1)	254	11.4^‡ ^(12.6)	248	11.3^‡ ^(13.0)	246	12.0^‡ ^(14.7)

Abbreviations: UBSE, upper-body strength and endurance; HG, hand grip; DASH, Disability of the Arm Shoulder and Hand questionnaire (0-100 scale, lower score = better function); SD, standard deviation.

* Results presented have been appropriately weighted (< 50 years:1.0; > 50 years:1.3) for oversampling of younger women.

^† ^Flexibility relates to flexion of the shoulder.

^‡ ^Statistically significant difference p < 0.05 from six months post-surgery.

**Table 2:** Proportion of women whose upper-body function declined, showed no change or improved between six- and 18-months post-surgery*

	Change in upper-body function from six- to 18-months post-surgery					
	Declined		No change = 0	Improved		Total
	n (%)	Median Change (Min, Max)	n (%)	n (%)	Median Change (Min, Max)	n (%)
*Objective measures*						
UBSE (kg)	41 (22.8)	-0.5 (-1.0, -0.25)	60 (32.9)	82 (44.3)	+0.5 (0.25, 2.5)	183 (100.0)
HG Strength (kg)	76 (41.6)	-3.1 (-14.0, -0.5)	10 (5.7)	99 (52.7)	+4.0 (0.5, 15.0)	185 (100.0)
Flexibility^† ^(degrees)	35 (17.7)	-10.0 (-45.0,-3.0)	39 (20.1)	119 (62.1)	+10.0 (5.0, 40.0)	193 (100.0)
*Self-reported measures*						
DASH score	76 (33.8)	+4.2 (0.6, 48.3)	22 (9.7)	134 (56.6)	-5.0 (-43.3, -0.7)	232 (100.0)

Abbreviations: UBSE, upper-body strength and endurance; HG, hand grip; DASH, Disability of the Arm Shoulder and Hand questionnaire (0-100 scale, lower score = better function).

* Results have been appropriately weighted (< 50 years:1.0; > 50 years:1.3) for oversampling of younger women.

^† ^Flexibility relates to flexion of the shoulder.

### Declines in upper-body function between six- and 18-months post-surgery

Adjusted relationships between personal and treatment characteristics and odds of decline in UBF between six- and 18-months PS are presented in Table 3. Older age, lower socioeconomic status as defined by income, being treated on the dominant side, having a mastectomy and more extensive lymph node surgery were associated with 2- to 3.5-fold increased odds of decline in objective UBF, with the relationships supported statistically (p < 0.05) except for having a mastectomy and extent of lymph node removal. Lower socioeconomic status, being treated on the dominant side and more extensive lymph node removal were also associated with increased odds of self-reported UBF declines (OR: 2.2-5.4; p < 0.05). In addition, older age was statistically but not clinically (OR: 1.9, p = 0.05) associated with increased odds of self-reported UBF declines, while having lymphoedema was clinically but not statistically associated with declines (OR: 2.4, p = 0.21).

**Table 3:** Personal and treatment-related characteristics associated with declines in upper-body function between six- and 18-months post-surgery*

	Odds of a Decline in UBSE Decline n = 41 (22.8%)				Odds of a Decline in DASH Decline n = 76 (33.8%)			
**Characteristics**^†^	n	Bivariate OR	Adjusted OR (95% CI)	p-value	n	Bivariate OR	Adjusted OR (95% CI)	p-value
Age				0.03				0.05
< 50	61	1.00	1.00 ref		80	1.00	1.00 ref	
50+	122	1.48	2.81 (1.14, 6.97)		152	2.35	1.94 (1.01, 3.75)	
Income				0.04				0.03
> $52,000 (include miss)	88	1.00	1.00 ref		104	1.00	1.00 ref	
$26,000-$51,999	50	0.90	1.76 (0.67, 4.60)		65	1.71	1.90 (0.98, 3.68)	
< $26,000	45	1.52	3.31 (1.29, 8.53)		63	2.47	2.46 (1.25, 4.85)	
Treated on Dominant Side				0.01				0.01
No	88	1.00	1.00 ref		118	1.00	1.00 ref	
Yes	95	2.40	2.56 (1.20, 5.42)		114	1.74	2.15 (1.24, 3.73)	
Surgery				0.06				
CLE	138	1.00	1.00 ref					
Mastectomy	45	1.95	2.25 (0.98, 5.21)					
Lymph Nodes Removed				0.35				0.02
None	24	1.00	1.00 ref		33	1.00	1.00 ref	
1 to 19	137	3.38	2.93 (0.68, 12.67)		166	2.22	2.63 (1.07, 6.45)	
20+	22	2.77	2.65 (0.43, 16.38)		33	2.69	4.81 (1.64, 14.14)	
Lymphoedema								0.21
No					154	1.00	1.00 ref	
Yes					18	1.73	2.41 (0.90, 6.44)	
Missing					60	1.27	1.15 (0.62, 2.11)	

Abbreviations: UBSE, upper-body strength and endurance; DASH, Disability of the Arm, Shoulder and Hand questionnaire (0-100 scale, lower score = better function); CI, Confidence Interval; ref, referent category against which all others are compared.

* Results have been appropriately weighted (< 50 years:1.0; > 50 years:1.3) for over sampling of younger women and adjusted for baseline upper-body function values.

^† ^All characteristics measured at baseline, six-months post-diagnosis.

### Objective and self-reported upper-body function and quality of life

Cross-sectional analyses using data from six- and 18-months PS demonstrate that there is a strong, inverse association between self-reported UBF (lower scores indicate better function) and QoL (r = -0.7 at each time, p < 0.01), but weak linear association between objective UBF and QoL (r = 0.18, p < 0.05 at six-months PS; r = 0.1, p = 0.12 at 18-months PS). Table 4 presents results of linear regression analyses exploring the relationships between change in UBF (from six- to 18-months PS) and QoL at 18-months PS, adjusting for baseline UBF as well as potential confounders identified elsewhere [24]. Declines in UBF between six- and 18-months PS were associated with lower QoL at 18-months PS, but the association was only clinically and statistically significant for self-reported UBF (greater than 7 unit difference between decliners and improvers, p < 0.01).

**Table 4:** Linear regression analyses of the relationships between changes in upper-body function (self-reported and objective) between six- and 18-months post-surgery and quality of life at 18-months post-surgery*

	Quality of life (FACTB+4) at 18-months post-surgery			
	n	Bivariate Mean	**Adjusted**^†^ Mean (95% CI)	p-value
*Objective upper-body function*				
UBSE change^‡^				0.10
declined	40	128.7	123.4 (118.5, 128.2)	
no change	60	132.6	127.9 (123.6, 132.2)	
improved	78	132.4	129.1 (125.2, 133.0)	

*Self-reported upper-body function*				
DASH change^‡^				< 0.01
declined	75	125.3	121.5 (118.2, 124.9)	
no change	22	145.1	127.8 (122.5, 133.2)	
improved	133	130.7	130.4 (127.5, 133.3)	

Abbreviations: FACTB+4, quality of life as measured by the Functional Assessment of Cancer Therapy Questionnaire (0 to 160 scale, higher score = better quality of life); UBSE, upper-body strength and endurance; DASH, Disability of the Arm, Shoulder and Hand questionnaire (0-100 scale, lower score = better function); CI, confidence intervals.

* Results presented have been appropriately weighted (< 50 years:1.0; > 50 years:1.3) for over-sampling younger women.

^† ^Means adjusted for all variables in table along with age, marital status, type of surgery, adjuvant therapy, number of co-morbidities, perceived handling of stress, baseline quality of life and baseline UBSE and DASH scores [24].

^‡ ^Change scores from six- to 18-months post-surgery, no change defined as 0.

## Discussion

Upper-body morbidity is common following treatment for breast cancer despite advances in treatment methods that have led to less invasive surgical techniques, such as sentinel node biopsy, and more refined, targeted radiation methods. While, on average, UBF improves between six- and 18-months PS, average change in UBF obscures substantial variation among individuals. Up to 40% (and no less than 17%) of women experienced declines in function during the 12-month study period. That is, even using six months following breast cancer surgery as baseline (rather than pre-diagnosis measures, which were unavailable for this study), further declines in UBF still occurred. Moreover, both objectively and subjectively measured UBF and declines in UBF have important implications for reported QoL.

**Figure 1:**
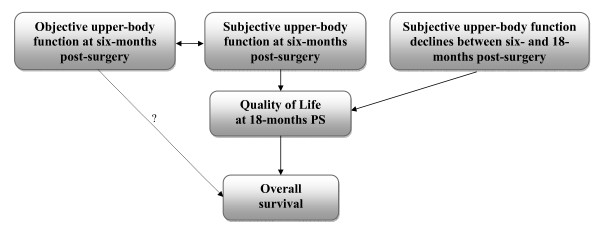
**Proposed relationships between upper-body function, quality of life and survival**.

Based on this and earlier work, the inter-relationships between UBF and QoL are emerging. Our results indicate that the relationships between objectively measured UBF and QoL post-surgery are minimal. In contrast, perceived UBF at both baseline and 18 months post-surgery revealed strong associations with QoL at the 18-month assessment, as was the association between perceived declines in UBF from six to 18 months post-surgery and QoL at 18 months post-surgery. That is, better perceived function and larger improvements in perceived function were associated with higher QoL. These findings are important for two reasons. First, we [25] and others [20] have reported that QoL can predict survival among women with breast cancer; hence, improving QoL has multiple benefits. Second, the modest correlation between objective and perceived UBF reported elsewhere [15] suggests that any intervention to improve UBF will need to attend to women's expectations as well as their physical function. Figure 1 provides a conceptual framework that could be tested, and developed further, in future longitudinal studies involving a larger breast cancer cohort than ours.

Given the potential clinical significance of UBF following breast cancer diagnosis, we sought to identify personal or behavioural characteristics that increase the likelihood of declining UBF. Earlier work by our group explored the relationship between treatment on the dominant side and UBF, and found that the strength of the relationship was as strong as that found between extent of lymph node removal (an established risk factor) and UBF [15]. Age, presence of co-morbidities and lower socioeconomic status also have been reported to influence arm function [15,26].

Older age, lower socioeconomic status, treatment on the dominant side and/or more extensive surgery to the chest wall or axilla were each independently associated with experiencing declines in UBF between six- and 18-months PS in this study. There also was evidence that having lymphoedema was associated with declines in perceived function during this period. The relationship between UBF declines and treatment on the dominant side may reflect, at least in part, regression to the mean, since the dominant side is typically stronger (clinically and perceived) compared to the non-dominant side. It is also pertinent to highlight that these characteristics together explain less than one-third of the variance for declines in objective UBF and less than 15% of the variance for self-reported function. More work, involving a more comprehensive assessment of potential risk factors or improving our assessments of known risk factors, is required to better understand who is at risk of experiencing UBF declines. Nevertheless, these results provide some initial description of women with breast cancer who might benefit from targeted intervention, focusing on UBF.

## Conclusion

This was a longitudinal study, using a population-based, representative sample of women with breast cancer, with results describing cross-sectional and predictive relationships between UBF and QoL. It is evident that declines in UBF continue to occur for some women well beyond the treatment period and that optimal UBF in the short- and longer-term following breast cancer is important with respect to concurrent QoL and subsequent QoL. Consequently, these findings provide support for the integration of a rehabilitation program into the care of women with breast cancer, which not only targets minimising declines and facilitating recovery during and following breast cancer treatment, but also assists women to optimise clinical function and come to terms with perceived changes that have occurred with respect to UBF. Given the extensive physical and psychosocial benefits that are known to occur with physical activity during and following breast cancer treatment [27], it seems plausible that a rehabilitation program with an emphasis on helping women become and/or stay active throughout their breast cancer experience would assist in this regard.

## Abbreviations

CI: confidence interval; DASH: Disability of the Arm Shoulder and Hand questionnaire; FACT-B+4: Functional Assessment of Cancer Therapy-Breast; HGS: hand grip strength; OR: odds ratio; PS: post-surgery; QoL: quality of life; SD: standard deviation; UBF: upper-body function; UBSE: upper-body strength and endurance.

## Competing interests

The authors declare that they have no competing interests.

## Authors' contributions

SCH supervised data collection and contributed to analysis, data interpretation and manuscript writing. SR and TD carried out analysis and contributed to manuscript writing. DB and BN provided critical input in data interpretation and manuscript writing. All authors read and approved the final manuscript.
